# Updated Meta-analysis on the Closure of Patent Foramen Ovale in Reduction of Stroke Rates: the DEFENSE-PFO Trial Does not Change the Scenario

**DOI:** 10.21470/1678-9741-2018-0194

**Published:** 2018

**Authors:** Michel Pompeu Barros Oliveira Sá, Erik Everton Silva Vieira, Luiz Rafael Pereira Cavalcanti, Roberto Gouveia Silva Diniz, Sérgio da Costa Rayol, Alexandre Motta de Menezes, Ricardo Felipe de Albuquerque Lins, Ricardo Carvalho Lima

**Affiliations:** 1 Department of Cardiovascular Surgery of the Pronto Socorro Cardiológico de Pernambuco (PROCAPE), Recife, PE, Brazil.; 2 Universidade de Pernambuco (UPE), Recife, PE, Brazil.; 3 Nucleus of Postgraduate Studies and Research in Health Sciences of the Faculdade de Ciências Médicas and Instituto de Ciências Biológicas (FCM/ICB), Recife, PE, Brazil.; 4 The CASUAL Investigators – CArdiovascular SUgery Academic League of the Universidade de Pernambuco (UPE), Recife, PE, Brazil.

**Keywords:** Foramen Ovale, Patent, Vascular Closure Devices, Meta-analysis

## Abstract

**Objective:**

We aimed to analyze whether patent foramen ovale (PFO) closure reduces the risk of stroke, assessing also some safety outcomes after the publication of a new trial.

**Introduction:**

The clinical benefit of closing a PFO has been an open question, so it is necessary to review the current state of published medical data in regards to this subject.

**Methods:**

MEDLINE, EMBASE, CENTRAL/CCTR, SciELO, LILACS, Google Scholar and reference lists of relevant articles were used to search for randomized controlled trials (RCTs) that reported any of the following outcomes: stroke, death, major bleeding or atrial fibrillation. Six studies fulfilled our eligibility criteria and included 3560 patients (1889 for PFO closure and 1671 for medical therapy.

**Results:**

The risk ration (RR) for stroke in the “closure” group compared with the “medical therapy” showed a statistically significant difference between the groups, favouring the “closure” group (RR 0.366; 95%CI 0.171–0.782, *P*=0.010). There was no statistically significant difference between the groups regarding the safety outcomes, death and major bleeding, but we observed an increase in the risk of atrial fibrillation in the “closure” group (RR 4.131; 95%CI 2.293–7.443, *P*<0.001). We also observed that the larger the proportion of effective closure, the lower the risk of stroke.

**Conclusion:**

This meta-analysis found that stroke rates are lower with percutaneously implanted device closure than with medical therapy alone, being these rates modulated by the rates of hypertension, atrial septal aneurysm and effective closure. The publication of a new trial did not change the scenario in the medical literature.

**Table:** 

Abbreviations, acronyms & symbols	
AHA	= American Heart Association
ASA	= American Stroke Association
CI	= Confidence interval
PFO	= Patent foramen ovale
PICOS	= Population, Intervention, Comparison, Outcome and Study design
PRISMA	= Preferred Reporting Items for Systematic Reviews and Meta-Analyses
RCTs	= Randomized controlled trials
RR	= Risk ration

## INTRODUCTION

### Rationale

Current American Heart Association;American Stroke Association (AHA;ASA) guidelines do not support the use of patent foramen ovale (PFO) closure among patients with PFO and cryptogenic stroke^[1]^. However, new meta-analysis of randomized controlled trials (RTCs)with the same number of patients and studies were published^[2-12]^ this year, all of them coming to the same conclusion: stroke rates are lower with percutaneously implanted device closure than with medical therapy alone. As we know, the medical literature currently changes at a fast pace. No sooner had all these meta-analyses been published than a new trial (DEFENSE-PFO) came out. Therefore, it is necessary to constantly review the current published medical data with regard to this subject.

### Objective

We aimed to analyze whether PFO closure reduces the risk of stroke, assessing also some safety outcomes. This analysis was planned in accordance with current guidelines for performing comprehensive systematic reviews and meta-analysis with meta-regression, including the Preferred Reporting Items for Systematic Reviews and Meta-Analyses (PRISMA)^[13]^ guidelines for RCTs. We pre-specified our analytical plan and registered the study protocol with PROSPERO, the international prospective register of systematic reviews (CRD42018084583).

## METHODS

### Eligibility Criteria

With the Population, Intervention, Comparison, Outcome and Study desing (PICOS) strategy, studies were only considered if: 1) the population comprised patients with recent stroke or transient ischemic attack who had a PFO; 2) there was an intervention group of device closure; 3) there was a control group receiving medical therapy; 4) studied outcomes included any of the following: stroke, death, major bleeding, atrial fibrillation; 5) studies were RCTs.

### Information Sources

The following databases were used (until April 2018): MEDLINE; EMBASE; CENTRAL/CCTR (Cochrane Controlled Trials Register); ClinicalTrials.gov; SciELO (Scientific Electronic Library Online); LILACS (Literatura Latino Americana em Ciências da Saúde); Google Scholar; and reference lists of relevant articles.

### Search

We conducted the research with Medical Subject Headings (MeSH) terms (‘Foramen Ovale, Patent' OR ‘Patent Oval Foramen ‘ OR ‘Oval Foramen, Patent' OR ‘Patent Foramen Ovale') AND (‘Stroke' OR ‘Cerebrovascular Accident' OR ‘Cerebrovascular Accidents' OR ‘CVA' OR ‘CVAs' OR ‘Cerebrovascular Apoplexy' OR ‘Apoplexy, Cerebrovascular' OR ‘Vascular Accident, Brain' OR ‘Brain Vascular Accident ‘ OR ‘Brain Vascular Accidents' OR ‘Vascular Accidents, Brain ‘ OR ‘Cerebrovascular Stroke' OR ‘Cerebrovascular Strokes' OR ‘Stroke, Cerebrovascular' OR ‘Strokes, Cerebrovascular' OR ‘Apoplexy ‘ OR ‘Cerebral Stroke' OR ‘Cerebral Strokes' OR ‘Stroke, Cerebral' OR ‘Strokes, Cerebral' OR ‘Stroke, Acute' OR ‘Acute Stroke' OR ‘Acute Strokes' OR ‘Strokes, Acute' OR ‘Cerebrovascular Accident, Acute' OR ‘Acute Cerebrovascular Accident' OR ‘Acute Cerebrovascular Accidents' OR ‘Cerebrovascular Accidents, Acute').

### Study Selection

The following steps were taken: 1) identification of titles of records through database research; 2) removal of duplicates; 3) screening and selection of abstracts; 4) assessment for eligibility through full-text articles; and 5) final inclusion in the study. One reviewer followed steps 1 to 3. Two independent reviewers followed step 4 and selected studies. The inclusion or exclusion of studies was decided unanimously. When there was disagreement, a third reviewer made the final decision.

### Data Items

The crude endpoints were stroke, death (any cause), major bleeding and atrial fibrillation.

### Data Collection Process

Two independent reviewers extracted the data. When there was disagreement about data, a third reviewer checked the data and made the final decision. From each study, we extracted patient characteristics, study design and outcomes.

### Risk of Bias in Individual Studies

Included studies were assessed for the following characteristics: sequence generation (randomization); allocation concealment (selection bias); blinding of participants and personnel (performance bias); blinding of outcome assessors (detection bias); incomplete outcome data addressed (attrition bias) and selective outcome reporting (reporting bias). Taking these characteristics into account, the papers were classified into A (low risk of bias), B (moderate risk of bias) or C (high risk of bias). Two independent reviewers assessed risk of bias. Agreement between the two reviewers was assessed with Kappa statistics for full-text screening and rating of relevance and risk of bias. When there was disagreement on risk of bias, a third reviewer checked the data and made the final decision.

### Summary Measures

The principal summary measures were RR with 95% confidence interval (CI) and *P* values (considered statistically significant when* P*<0.05) for stroke, death, major bleeding and atrial fibrillation. The meta-analysis was completed with the Comprehensive Meta-Analysis software (version 2, Biostat, Inc., Englewood, NJ, USA).

### Synthesis of Results

Forest plots were generated for graphical presentations of clinical outcomes, and we performed the I^2^ test and χ^2^ test for the assessment of heterogeneity across the studies^[14]^. Inter-study heterogeneity was explored using the χ^2^ statistic, but the I^2^-value was calculated to quantify the degree of heterogeneity across the studies that could not be attributable to chance alone. When I^2^ was more than 50%, significant statistical heterogeneity was considered to be present. Each study was summarized by the difference in means or RR, depending on the analyzed outcome. The RR and the differences in means were combined across studies using a weighted DerSimonian–Laird random effects model^[15]^.

### Risk of Bias Across Studies 

To assess publication bias, a funnel plot was generated for each outcome, statistically assessed by Begg and Mazumdar's test^[16]^ and Egger's test^[17]^.

### Sensitivity Analysis

We analyzed the pool data regarding the outcome “stroke” according to the presence (or absence) of atrial septal aneurysm.

### Meta-Regression Analysis

Meta-regression analysis was performed to determine whether the effects of the PFO closure were modulated by pre-specified factors. Meta-regression graphs describe the effect of aspirin on the outcome (plotted on the y-axis) as a function of a given factor (plotted as a mean or proportion of that factor on the x-axis). Meta-regression coefficients show the estimated increase in log risk ration (RR) per unit increase in the covariate. Since log RR > 0 corresponds to RR > 1 and log RR < 0 corresponds to RR < 1, a negative coefficient would indicate that as a given factor increases, the RR decreases, and vice versa.

The pre-determined modulating factors examined were: age (mean – years), male gender (%), hypertension (%), smoking (%), large shunt before the interventions, atrial septal aneurysm and effective closure (freedom from large shunt after the interventions).

## RESULTS

### Study Selection

**Fig. 1:**
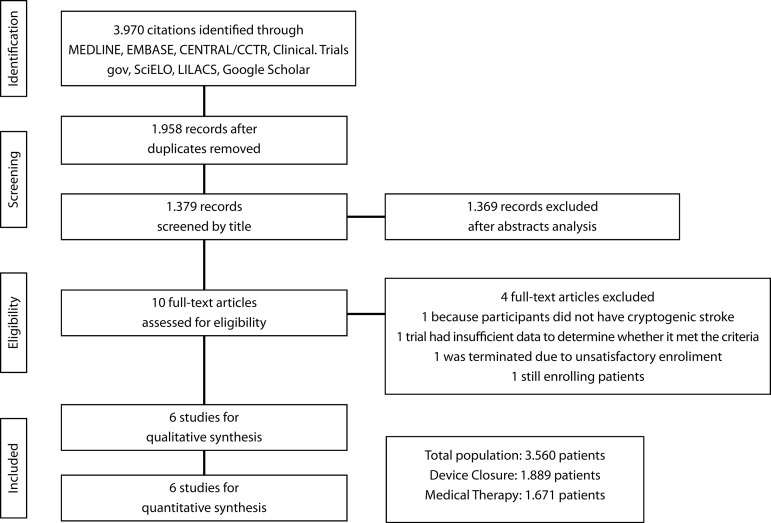
Flow Diagram of Studies Included in Data Search.

A total of 3,970 citations were identified, of which 10 studies were potentially relevant and retrieved as full-text. Six^[18-23]^ publications fulfilled our eligibility criteria. Interobserver reliability of study relevance was excellent (Kappa=0.82). Agreement for decisions related to study validity was very good (Kappa=0.84). The search strategy can be seen in Figure 1.

### Study Characteristics

A total of 3,560 patients (device closure: 1,889 patients; medical therapy: 1,671 patients) were included from studies published from 2012 to 2018. All the trials were multicentric. Most studies consisted of patients whose mean or median age was approximately on the fourth decade of life. The medical therapy in the studies was not homogeneous, since different regimens were applied (aspirin, clopidogrel, dipyridamole, combined regimens, etc). The same goes for the devices used, being the CLOSE trial most noteworthy for applying various devices (Table 1). The overall internal validity was considered “low risk of bias” (Table 2).

**Table 1:** Characteristics of populations.

	DEFENSE-PFO (N=120)	CLOSE (N=473)	REDUCE (N= 664)	PC (N=414)	RESPECT (N=980)	CLOSURE (N=909)
**% of data in meta-analysis**	3.3	13.3	18.7	11.6	27.5	25.5
**Demographic variables**						
Age ± SD, years	49.0±15.0	43.3±10.3	45.1±9.45	44.5±10.2	45.4±9.8	45.5±10.2
Male (%)	55.8	58.9	60.1	49.8	54.7	51.8
**Medical history variables**						
Currently smoking (%)	21.7	28.9	13.3	23.9	13.3	15.2
Coronary artery disease (%)	NR	NR	NR	1.9	2.9	2.1
Diabetes (%)	11.7	2.5	4.2	2.6	7.4	7.8
Hypercholesterolemia (%)	35.8	13.9	NR	27.1	39.5	44.1
Hypertension (%)	32.5	10.7	25.6	25.8	31.4	31.0
Migraine (%)	NR	30.6	NR	20.5	38.8	33.6
Prior stroke/TIA (%)	NR	3.6	85	37.4	18.6	12.5
**Echocardiographic variables**						
Atrial septal aneurysm (%)	10.8	32.7	NR	23.7	35.6	35.6
Large shunt (%)	57.5	92.8	39.3	21.7	76.1	61.1
**Treatment variables**						
Randomized to device closure(%)	50.0	50.3	66.4	49.3	50.9	49.2
Treated with medical therapy (%)	50.0	49.6	33.6	80.0	88.0	84.7
Device	Amplatzer PFO Occluder (St. Jude Medical)	Amplatzer PFO Occluder or Cribriform; Starflex; CardioSeal; Intrasept PFO; PFOStar; Helex; Premere; PFO occluder OCCLUTECH; PFO occluder GORE (GSO)	EITHER the Helex Septal Occluder device OR the Cardioform Septal Occluder	Amplatzer PFO Occluder (St. Jude Medical)	Amplatzer PFO Occluder (disc occluder)	STARFlex septal closure system (umbrella occluder)

**Table 2:** Analysis of Risk of Bias: Internal Validity.

Study	Randomization	Selection bias	Performance bias	Detection bias	Attrition bias	Reporting bias
DEFENSE-PFO	A	B	B	A	A	A
CLOSE 2017	A	A	B	A	A	A
REDUCE 2017	A	A	B	A	A	A
RESPECT 2013	A	A	A	A	A	A
PC 2013	A	A	A	A	A	A
CLOSURE I 2012	A	A	A	A	A	A

A=risk of bias is low; B=risk of bias is moderate; C=risk of bias is high; D=incomplete reporting

### Synthesis of Results

**Fig. 2:**
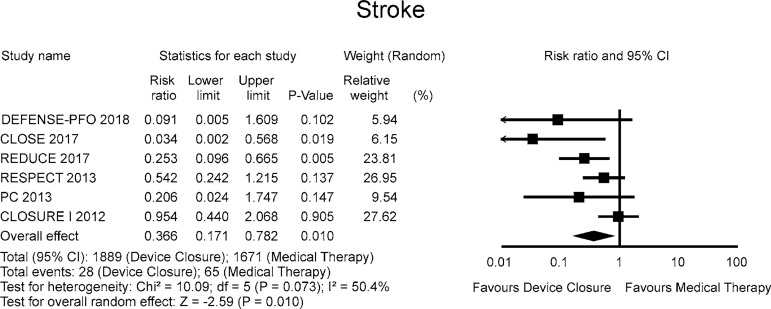
Forest Plots of Efficacy Outcomes.

The RR for stroke in the “device closure” group compared with the “medical therapy” group in each study is reported in Figure 2. There was evidence of moderate heterogeneity of treatment effect among the studies for stroke. The overall RR (95% CI) of stroke showed a statistically significant difference between the groups, favouring the “device closure” group (random effect model: RR 0.366; 95%CI 0.171 – 0.782, *P*=0.010).

**Fig. 3:**
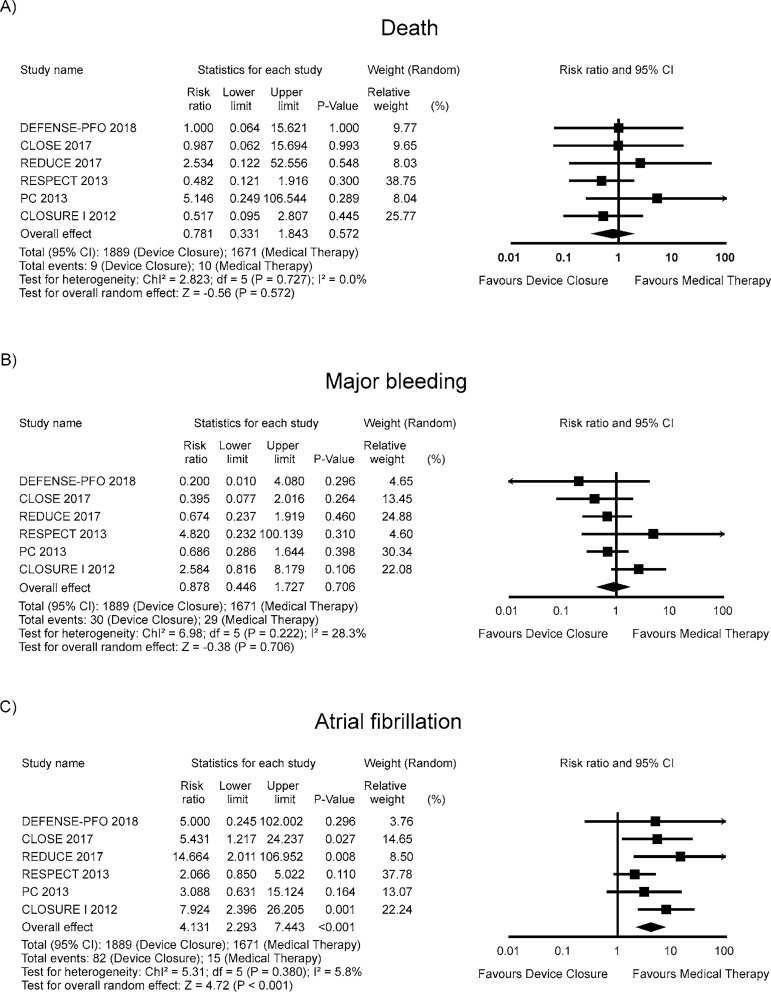
Forest Plots of Safety Outcomes.

The RR for death in the “device closure” group compared with the “medical therapy” group in each study is reported in Figure 3A. There was no evidence of heterogeneity of treatment effect among the studies for death. The overall RR (95% CI) of death showed no statistically significant difference between the groups (random effect model: RR 0.781; 95%CI 0.331 – 1.843, *P*=0.572).

The RR for major bleeding in the “device closure” group compared with the “medical therapy” group in each study is reported in Figure 3B. There was evidence of mild heterogeneity of treatment effect among the studies for major bleeding. The overall RR (95% CI) of major bleeding showed no statistically significant difference between the groups (random effect model: RR 0.878; 95%CI 0.446 – 1.727, *P*=0.706).

The RR for atrial fibrillation in the “device closure” group compared with the “medical therapy” group in each study is reported in Figure 3C. There was evidence of mild heterogeneity of treatment effect among the studies for atrial fibrillation. The overall RR (95% CI) of atrial fibrillation showed a statistically significant difference between the groups (random effect model: RR 4.131; 95%CI 2.293 – 7.443, *P*<0.001).

### Risk of Bias Across Studies

**Fig. 4:**
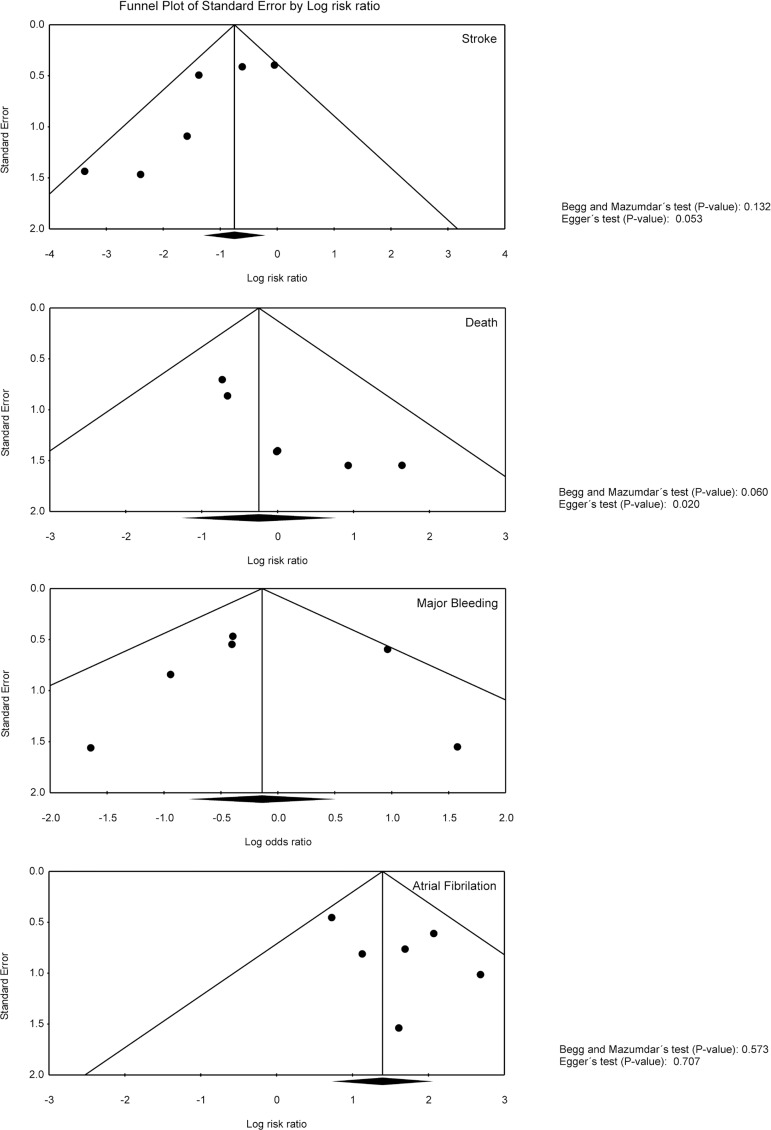
Publication Bias Analysis of Clinical Outcomes by Funnel Plot Graphic.

Funnel plot analysis (Figure 4) disclosed no asymmetry around the axis for the outcomes stroke, major bleeding and atrial fibrillation, which means that we have low risk of publication bias related to these outcomes. However, we detected a possibility of publication bias for the outcome death.

### Sensitivity Analysis

**Fig. 5:**
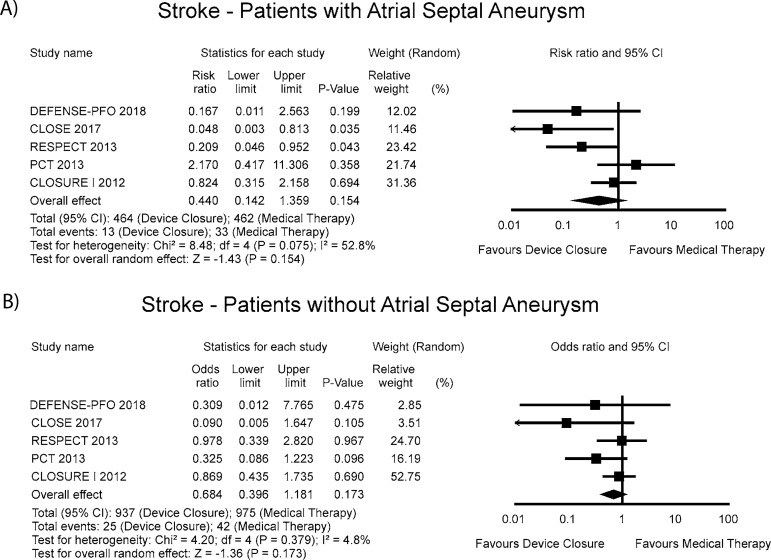
Sensitivity analysis for the presence of an atrial septal aneurysm.

Searching for evidence of a particular impact of the presence of an atrial septal aneurysm on the results, we detected no difference between the groups (Figure 5). Unfortunately, the REDUCE trial^[20]^ was left out of this last analysis because the presence of an atrial septal aneurysm was determined at the time of the PFO closure procedure and, therefore, it was not recorded before trial entry or among the patients in the antiplatelet-only group.

### Meta-Regression Analysis

**Fig. 6:**
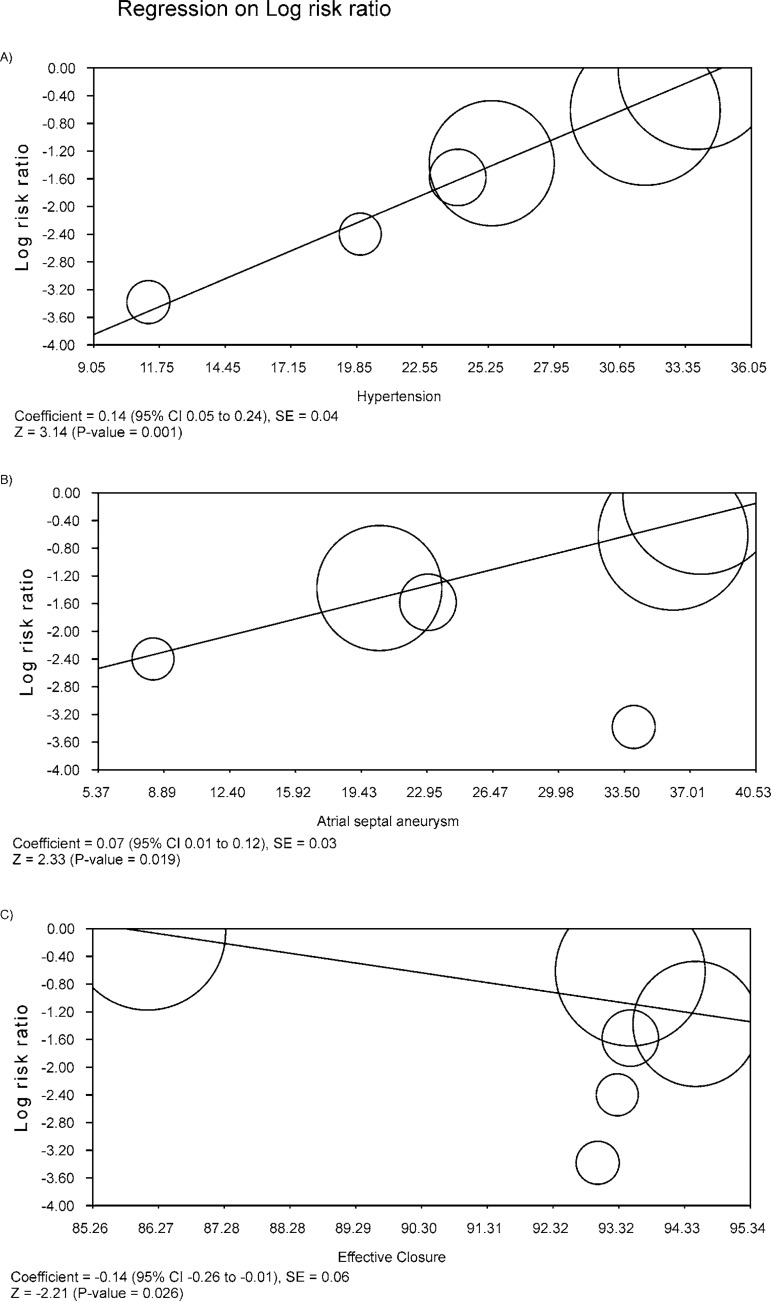
Meta-regression analysis.

Meta-regression coefficients were statistically significant for the variables hypertension, atrial septal aneurysm and effective closure regarding the outcome “stroke”. For the variables hypertension and atrial septal aneurysm, we observed that the larger the proportion of patients with hypertension and the larger the proportion of patients with atrial septal aneurysm, the higher the risk for stroke (Figures 6A, 6B). Conversely, the larger the proportion of effective closure, the lower the risk of stroke (Figure 6C).

## DISCUSSION

### Summary of Evidence

To our knowledge, this is the largest meta-analysis of studies performed to date that provides incremental value by demonstrating that patients seem to benefit from device closures in comparison to medical therapy in the reduction of the rate of stroke. On the other hand, there was an increase in the rates of atrial fibrillation. We did not identify the group of patients with an atrial septal aneurysm as a particular group that benefits from the device closure in the sensitivity analysis, although we identified this variable as a modulation factor of the risk for stroke in the meta-regression. We also observed that the benefit of the device closure in the reduction of the rates of stroke hinges on the rate of effective closure. We did not find evidence that the publication of the DEFENSE-PFO trial changed the scenario in the medical literature.

### Some Comments

The lack of efficacy observed in the CLOSURE I trial has been put down to ineffective PFO closure in the device arm, with 14% demonstrating significant residual right-to-left shunting, whereas, in the other trials, we observed the following rates: 3.3% (DEFENSE-PFO), 7% (CLOSE), 5.5% (REDUCE), 6.5% (RESPECT) and 6.5% (PC trial). Our meta-regression showed that the more successful the closure, the lower the risk of stroke in the device group (Figure 6C). Therefore, we must bare in mind that “procedural success”, which was defined in the studies as successful implantation with no complications, does not mean “success of PFO closure”, which was defined in the studies as minimal or no shunt after the procedure.

### Risk of Bias and Limitations of the Present Study

There are inherent limitations with meta-analyses, including the use of cumulative data from summary estimates. Patient data were gathered from published data, not from individual patient follow-up. Access to individual patient data would have enabled us to conduct further subgroup analysis and propensity analysis to account for differences between the treatment groups. This meta-analysis included only data from randomized studies, which do not reflect the “real world” but, on the other hand, are less limited by publication bias, treatment bias, confounders, and a certain tendency to overestimate treatment effects observed in the observational studies, since patient selection alters outcome and thus makes non-randomized studies less robust.

Moreover, besides statitiscal heterogeneity in some analyses, there is also the issue of the clinical heterogeneity that might have played some role in the pooled results. For instance, in the CLOSE trial, eleven different devices were appplied for PFO closure. In the antiplatelet-only group and the PFO closure group, 410 patients (86.7%) received aspirin, 51 (10.8%) received clopidogrel, 6 (1.3%) received aspirin with extended-release dipyridamole, and 6 (1.3%) received aspirin with clopidogrel. As we can see, not all of patients were 100% equally treated.

## CONCLUSION

This meta-analysis found that stroke rates are lower with percutaneously implanted device closure than with medical therapy alone, being these rates modulated by the rates of effective closure. The publication of the DEFENSE-PFO trial did not change the current scenario.

**Table:** 

Authors' roles & responsibilities	
MPBOS	Conception and design, analysis and interpretation of data, drafting of the manuscript, revising it critically for important intellectual content; final approval of the version to be published
EESV	Collection of data, drafting of the manuscript, revising it critically for important intellectual content; final approval of the version to be published
LRPC	Collection of data, drafting of the manuscript, revising it critically for important intellectual content; final approval of the version to be published
RGSD	Revising it critically for important intellectual content; final approval of the version to be published
SCR	Revising it critically for important intellectual content; final approval of the version to be published
AMM	Revising it critically for important intellectual content; final approval of the version to be published
RFAL	Revising it critically for important intellectual content; final approval of the version to be published
RCL	Revising it critically for important intellectual content; final approval of the version to be published
